# Liquefied capsules containing nanogrooved microdiscs and umbilical cord-derived cells for bone tissue engineering

**DOI:** 10.12688/openreseurope.17000.2

**Published:** 2024-09-09

**Authors:** Mariana Carreira, Manuel Pires-Santos, Clara R Correia, Sara Nadine, João F Mano

**Affiliations:** 1 CICECO – Aveiro Institute of Materials, Department of Chemistry, University of Aveiro, Aveiro, Aveiro District, 3810-193, Portugal

**Keywords:** 3D culture, bone regeneration, in vitro vascularization, nanotopography, stem cells, umbilical cord tissue, liquefied capsules

## Abstract

**Background:**

Surface topography has been shown to influence cell behavior and direct stromal cell differentiation into distinct lineages. Whereas this phenomenon has been verified in two-dimensional cultures, there is an urgent need for a thorough investigation of topography’s role within a three-dimensional (3D) environment, as it better replicates the natural cellular environment.

**Methods:**

A co-culture of Wharton’s jelly-derived mesenchymal stem/stromal cells (WJ-MSCs) and human umbilical vein endothelial cells (HUVECs) was encapsulated in a 3D system consisting of a permselective liquefied environment containing freely dispersed spherical microparticles (spheres) or nanogrooved microdiscs (microdiscs). Microdiscs presenting 358 ± 23 nm grooves and 944 ± 49 nm ridges were produced via nanoimprinting of spherical polycaprolactone microparticles between water-soluble polyvinyl alcohol counter molds of nanogrooved templates. Spheres and microdiscs were cultured *in vitro* with umbilical cord-derived cells in a basal or osteogenic medium within liquefied capsules for 21 days.

**Results:**

WJ-MSCs and HUVECs were successfully encapsulated within liquefied capsules containing spheres and microdiscs, ensuring high cellular viability. Results show an enhanced osteogenic differentiation in microdiscs compared to spheres, even in basal medium, evidenced by alkaline phosphatase activity and osteopontin expression.

**Conclusions:**

This work suggests that the topographical features present in microdiscs induce the osteogenic differentiation of adhered WJ-MSCs along the contact guidance, without additional differentiation factors. The developed 3D bioencapsulation system comprising topographical features might be suitable for bone tissue engineering approaches with minimum *in vitro* manipulation.

## Introduction

Bone tissue engineering (TE) has evolved remarkably in recent years. Taking advantage of the tools provided by materials science, it is now possible to manufacture implantable biomedical devices that recapitulate the naturally highly vascularized environment of bone. The scientific community dedicated to bone TE is proposing three-dimensional (3D) systems that integrate vascular features. The aim is to design 3D structures that simultaneously allow the deposition of a mineralized osteogenic-like extracellular matrix (ECM) while promoting the creation of a vascular network ^1– 6^. These bone-engineered systems should, once implanted, establish anastomosis with the host vasculature.

The late strategy in bone scaffold vascularization relies on the co-culture of mesenchymal stem/stromal cells (MSCs) and endothelial cells (ECs) ^7– 9^. In direct contact, these two cell types are known to communicate through paracrine stimulation and intercellular gap junctions ^10– 13^. MSCs and osteoblasts secrete vascular endothelial growth factor (VEGF), a potent angiogenic factor ^11, 12, 14, 15^. ECs secrete bone morphogenetic proteins (BMP), including BMP-2, a key factor required for osteogenic differentiation and initiation of bone repair ^16– 18^. Several studies reported that the co-culture of MSCs and ECs enhances the expression of early and late osteogenic markers, such as alkaline phosphatase (ALP) and osteocalcin, respectively ^8, 10, 19, 20^. Furthermore, MSCs were found to behave like perivascular and support the quiescence of endothelial cells and the stabilization of newly formed capillary networks ^21– 25^. MSCs can be isolated from multiple sources and have the ability to differentiate *in vitro* into the classic trilineage, namely osteogenic, adipogenic, and chondrogenic. Bone marrow-derived (BM) MSCs have been widely used as an osteogenic source for bone regeneration, however, its isolation is an invasive and painful procedure associated with patient morbidity and other related complications ^26, 27^. Moreover, MSCs are present at a very low frequency in bone marrow, corresponding to 0.001% to 0.01% of the BM nucleated cells, which requires time-consuming *in vitro* expansion ^28, 29^. It is also known that there is a reverse correlation between cell age, and proliferation and differentiation potential ^30^. Consequently, other sources are being explored, such as MSCs isolated from the Wharton’s jelly of the umbilical cord tissue (WJ-MSCs) ^31– 33^. Similar to BM-MSCs, WJ-MSCs are able to differentiate into the classical triple lineage and present immunomodulatory properties ^30^. In addition, these cells are harvested from tissues that are usually discarded, without surgical invasive procedures, thus avoiding major ethical issues. WJ-MSCs display a greater ability to expand in culture, with a faster doubling time when compared to BM-MSCs ^34^. Such ability could be related to their youth and the presence of longer telomeres. WJ-MSCs are suggested to express more genes related to stemness, growth, and angiogenesis compared to BM-MSCs, whereas the latter are more likely to express genes associated with bone development ^35– 37^. It was also reported greater ability of WJ-MSCs in the stimulation of microvascular structures relative to BM-MSCs ^36, 38^. This angiogenic potential is especially important for bone scaffold vascularization and implantation. Although WJ-MSCs are more primitive cells, as suggested by stemness-related genes and the preservation of embryonic stem cell markers, they may have advantageous properties in tissue regeneration ^39^. Nevertheless, both *in vitro* and *in vivo* potential of WJ-MSCs in TE requires a better understanding and investigation.

The umbilical cord (UC) tissue is also a source of a particular type of macrovascular cells, known as human umbilical vein endothelial cells (HUVECs). These mature ECs are widely used as *in vitro* models of angiogenesis and vascularized TE constructs ^8, 25, 40, 41^. From a bone tissue engineering perspective, the UC presents a promising opportunity as a source of cells as both MSCs and ECs can be isolated from the same donor tissue. The successful isolation of cells from a single tissue may facilitate the development of personalized and vascularized bone implants for each patient.

Apart from co-culture systems, the bone TE community has been exploring the potential of topographical surfaces on MSCs osteogenic differentiation through contact guidance ^42– 53^. It is also known that topography influences the EC's behavior ^54– 60^. However, most of these findings are associated with two-dimensional (2D) cultures, and only a few include co-cultures of MSCs and ECs, which do not accurately recapitulate the native bone environment ^54, 55, 61^. Moreover, the use of topographical cues is usually combined with the addition of supplemental osteogenic factors to the culture medium, namely dexamethasone, ascorbic acid, and β-glycerophosphate. In a recent study, our research group reported a nanotopography-driven full osteogenic differentiation in a 3D system, in the absence of additional osteogenic factors ^62^. Adipose-derived stem cells (ASCs) were encapsulated with nanogrooved microparticles in a liquefied core surrounded by a multilayered membrane. This type of liquefied and multilayered encapsulation system comprising microparticles has also been proven to support ASCs and ECs survival (e.g. signaling, proliferation), osteogenic differentiation, and bone matrix deposition ^63– 66^. Furthermore, the permeability of the multilayered membrane permitted the release of soluble factors produced by the encapsulated cells, such as VEGF, which is ultimately required for bone scaffold integration and the establishment of anastomosis within the host vasculature, once implanted.

Given the importance of co-cultures and the advantage of the use of UC-derived cells, we aim to explore the osteogenic differentiation potential driven by topographical cues in a 3D co-culture of WJ-MSCs and HUVECs. The novelty of this study lies in the use of only cells isolated from the UC tissue. To truly evaluate and distinguish the topographical effect itself in the process of bone formation *in vitro*, we aim to assess the osteogenic differentiation potential of topographic particles in the absence of the three classical supplemental osteogenic differentiation factors. Here, we propose a 3D autonomous system with minimal *in vitro* manipulation, where WJ-MSCs can differentiate into osteoblasts through direct contact with HUVECs, and contact guidance provided by a nanogrooved pattern. This innovative engineered 3D bioencapsulation system is composed of (i) a multilayered membrane assembled by layer-by-layer (LbL) deposition, (ii) a liquefied core containing nanogrooved microdiscs and (iii) a co-culture of WJ-MSCs and HUVECs. Since both WJ-MCSs and HUVECs are anchorage-dependent cells, we added spherical microparticles to provide adhesion support to cells in the control group.

We hypothesized that within the privileged environment of liquefied and multilayered capsules, the contact with the HUVECs and the geometrical cues provided by the nanogrooved microdiscs will promote the osteogenic differentiation of the WJ-MSCs, and ultimately lead to the creation of an *in vitro* mineralized and vascularized bone-like microtissue.

## Methods

### Cell isolation and characterization

MSCs and HUVECs were isolated from two separated human UC of newborn babies. The collected tissues were obtained under a cooperation agreement between the CICECO - Aveiro Institute of Materials - University of Aveiro and Hospital do Baixo Vouga (Aveiro, Portugal) after approval of the Competent Ethics Committee (CEC) dated 20 ^th^ October 2020. The received human tissues were handled under the guidelines approved by the CEC. Written informed consent was obtained from all subjects. The samples were collected in a container with Dulbecco′s phosphate- buffered saline solution (DPBS, pH 7.4–7.6, Gibco) supplemented with 1% (v/v) antibiotic/antimycotic (ThermoFisher Scientific) and kept at 4°C until the isolation procedure. The samples were transferred to the laboratory facilities within 24 h after collection and immediately processed. The UCs were washed several times with sterile DPBS to remove blood and blood clots. MSCs were isolated from the Wharton’s jelly part of the UC by the explant method and cultured at 37 °C and 5% of CO _2_ atmosphere in Minimum Essential Medium Alpha (α-MEM, Gibco) supplemented with 1% (v/v) antibiotic/antimycotic and 10% (v/v) heat-inactivated fetal bovine serum (FBS, Gibco). Cells were maintained in culture until passage 3, and the medium was changed twice a week. HUVECs were dissociated from the UC vein wall by enzymatic digestion using 0.1% (w/v) collagenase type IA (MP Biomedicals, USA). Cells were maintained in culture flasks coated with 0.7% (w/v) gelatin (type A, from porcine skin, ~300 g bloom, Sigma-Aldrich) at 37 °C and 5% of CO _2 _atmosphere in M199 medium (Sigma-Aldrich) supplemented with 1% (v/v) of endothelial cell growth supplement (ECGS, 40 mg.mL ^-1^, Merck, Germany), 10% (v/v) of heparin (100 mg.mL ^-1^, Sigma-Aldrich), 20% (v/v) FBS, 1% (v/v) antibiotic/antimycotic until passage 2. The culture medium was changed twice a week. Cell phenotype characterization was performed by flow cytometry (Flow Cytometry BD Accuri C6 Plus). Briefly, WJ-MSCs and HUVECs were dissociated with triple express (TrypLE™ Express Enzyme (1X), phenol red, Thermo Fisher Scientific) and resuspended in a staining/washing solution containing 2% (w/v) bovine serum albumin (BSA, Sigma-Aldrich) and 0.1% (w/v) sodium azide (TCI) prepared in DPBS. MSCs were incubated with the monoclonal antibodies PE-conjugated CD73, AlexaFluor 647-conjugated CD90 and AlexaFluor 488-conjugated CD105 for 45 min at 4 °C protected from light. Incubation with FITC-conjugated CD34 and APC-conjugated CD31 was performed as negative markers. HUVECs were incubated for 45 min at 4 °C in the dark with the monoclonal antibodies APC-conjugated CD31; incubation with FITC-conjugated CD34 was used a negative marker. All antibodies were purchased from BioLegend (1 mg.mL ^-1^) and used 1:20 diluted. Samples were washed in the washing/staining solution, and subsequently resuspended in the acquisition buffer solution containing 1% (v/v) formaldehyde (Sigma-Aldrich) and 0.1% (w/v) sodium azide in PBS. Samples were stored at 4 °C until analysis.

### Polycaprolactone microparticles production and surface functionalization

Spherical polycaprolactone (PCL) microparticles were produced by emulsion solvent evaporation technique, as described elsewhere ^62, 65, 67^. Briefly, a 5% (w/v) solution of PCL (Mn=80 kDa, Sigma-Aldrich) dissolved in dichloromethane (DCM, Labsolve), was added dropwise using a 21 G needle to a 0.5% (w/v) polyvinyl alcohol (PVA, Mn=30 – 70 kDa, Sigma-Aldrich) solution dissolved in distilled water, under agitation. The resulting solution was left under stirring for 2 days at room temperature (RT) to evaporate the organic solvent. The formed spherical microparticles (spheres) were washed several times in distilled water and separated by size using sieves (HAVER Test Sieve with stainless steel frame 50 mm × 25 mm). After sieving, spheres within the range of 25 to 40 μm and 80 to 100 μm (diameter) were washed in distilled water, collected in absolute ethanol (ethanol absolute anhydrous, Carlo Erba), and were left to dry in the oven at 37 °C for one week. To evaluate the size distribution of the spherical microparticles within the range of 80 to 100 µm, spheres were visualized under the microscope (Microscope ZEISS Primo Star HAL/LED), and their diameter (n=50) was measured using the Image J software. The size distribution of the spherical microparticles within the range of 25 to 40 µm is already described ^62^. Spheres within 25 to 40 μm were used to produce nanogrooved microdiscs (microdiscs). For that, PVA was dissolved in distilled water under agitation at 90 °C to prepare a 12% (w/v) PVA solution. This solution was poured onto clean optical media substrates (CDs) to produce patterned PVA molds, following a degassing step on a vacuum desiccator. PVA molds were dried at 40 °C in the oven for approximately 1 week and subsequently dissociated from the CDs. Spheres were sprinkled over a 140 μm sieve on the top of pattern PVA membranes and heated for 25 min in an oven at 75 °C. A second PVA mold was placed on top of the pre-heated particles with the pattern facing down. A pressure of approximately 130 mPa was applied for 5 min. Samples were maintained with the weight on top until the temperature cooled down below 50 °C. The resulting PVA-PCL-PVA “sandwiches” were carefully collected, without separation, and washed several times during 1 week in distilled water under stirring to dissolve the PVA molds. The produced nanogrooved microdiscs were collected in absolute ethanol and were left to dry in the oven at 37 °C for one week. Prior to cell culture, the surface of spheres and microdiscs was modified via plasma treatment (Plasma System ATTO, Electronic Diener) for 15 min at 0.4-0.6 mbar and 30 V of electrical potential difference. Samples were sterilized for 2 h in 70% (v/v) ethanol and washed in DPBS. The sterile PCL microparticles were coated with 10 µg.cm ^-2^ of collagen type I (collagen, type I solution from rat tail, Sigma-Aldrich) overnight at 4°C. Before use, spheres and microdiscs were well washed in DPBS.

### Production of liquefied and multilayered capsules

Sodium alginate (2% w/v, ALG, low viscosity from brown algae, Sigma-Aldrich) was dissolved in sodium chloride (0.15 M, NaCl, LABCHEM) buffered at pH 6.7 with MES hydrate (25 mM, Alfa Aesar). The solution was filtrated with a 0.22 μm syringe filter.

MSCs were expanded in T175 culture flasks (5×10 ^3^ cells.cm ^-2^) in α-MEM medium at 37 °C in a humidified atmosphere with 5% CO _2_ until 90% confluence. HUVECs were expanded in T175 culture flasks (5×10 ^3^ cells.cm ^-2^) coated with 0.7% (w/v) gelatin in M199 medium supplemented with 1% (v/v) of ECGS and 10% (v/v) of heparin at 37 °C in a humidified atmosphere with 5% of CO _2_ until reaching 90% confluence. The media changes were performed twice a week. WJ-MSCs and HUVECs were detached with 1× trypsin-EDTA (ThermoFisher Scientific) solution prepared in phosphate-buffered saline solution (PBS, Sigma-Aldrich), at 37 °C for 5 min. Cell suspensions were neutralized with culture medium and centrifugated for 5 min at 300 g. WJ-MSCs were used at passage 5 and HUVECs were used at passage 6.

Spheres and microdiscs were separately mixed with the alginate solution (20 mg per mL ALG) to produce two experimental conditions. WJ-MSCs and HUVECs (1:1) were added to the alginate solutions containing spheres or microdiscs (3.5 × 10 ^6^ cells per mL ALG).

**Figure 1.**
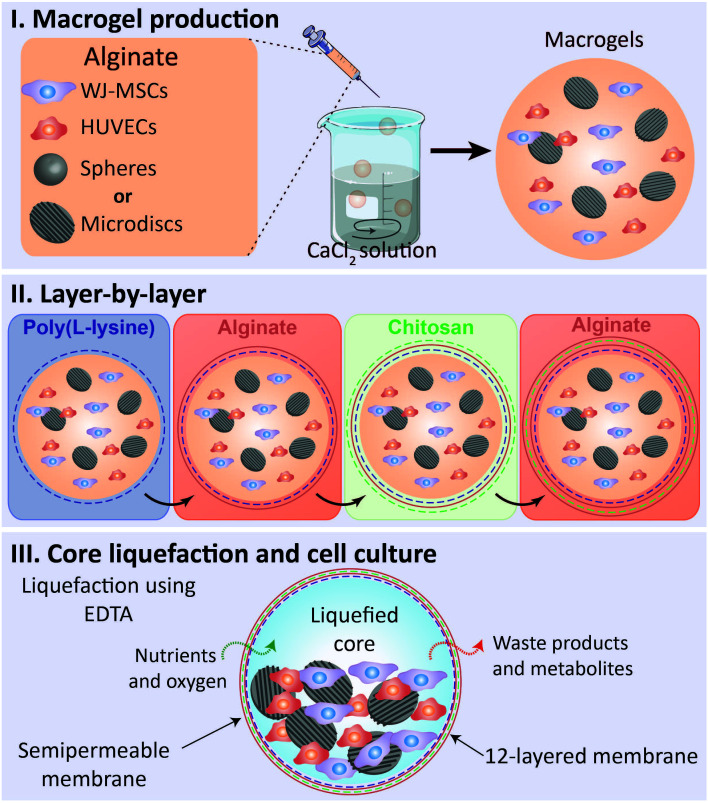
Methodology’s illustration – liquefied capsule production for co-culture of WJ-MSCs and HUVECS with spherical microparticles (spheres) or nanogrooved microdiscs (microdiscs). **I.** Macrodroplets of alginate containing WJ-MSCs, HUVECs, and spheres or microdiscs were dropwised into a calcium chloride solution. **II.** A 12-layered and semipermeable membrane was built around the formed macrogels through the layer-by-layer technique utilizing the following sequence of polyelectrolytes: poly(L-lysine), alginate, chitosan, and alginate. **III.** The sacrificial core liquefication was obtained by immersion in ethylenediaminetetraacetic acid solution (EDTA). Macrocapsules were maintained for 21 days in culture in a basal or osteogenic medium.

Under agitation, alginate solution containing cells and spheres or microdiscs were dropwised into a calcium chloride solution (0.1 M, CaCl _2_, Merck) buffered at pH 6.7 with MES hydrate (25 mM, Alfa Aesar) using a 21 G needle. Alginate droplets were immediately crosslinked by ionotropic gelation with calcium ions. The hydrogel macrodroplets formed were left to stir for 20 min at RT. Then, hydrogel droplets were collected and rinsed in a solution containing 0.15 M NaCl and 25 mM MES. Once the droplets loaded with cells and spheres or microdiscs were obtained, the external membrane was processed by LbL deposition. ALG capsules were immersed in poly(L-lysine) (PLL, Mw ~ 30.000 – 70.000, pH 6.7, Sigma-Aldrich), ALG solution (pH 6.7), and water-soluble chitosan (CHT, Protasan UP CL 213, viscosity 107 mPas, Mw=2.7×10 ^5^ g.mol ^-1^, 83% degree of deacetylation, pH 6.3, NovaMatrix), and in ALG again. A 10-min polyelectrolyte adsorption period was performed between each polymer with a 5 min washing step in NaCl/MES to remove the macromolecules in excess. This process was repeated three times to obtain a final 12-layered membrane coating. Ultimately, the capsule core was liquefied by immersion for 5 min in 0.02 M ethylenediaminetetraacetic acid solution (EDTA, pH 6.7, Sigma-Aldrich) dissolved in water for injection (WFI, ThermoFisher Scientific) containing 0.15 M NaCl and 25 mM MES. All the polyelectrolytes (0.5 mg.mol ^-1^) were dissolved in a solution containing 0.15 M NaCl and 25 mM MES solution. All the solutions used in the production of liquefied and multilayered capsules were sterilized by filtration with a 0.22 μm filter. The above information is represented in Figure 1.

### *In vitro* culture

The capsules containing cells and spheres or microdiscs were cultured for 21 days in basal medium, consisting of M199 medium supplemented with 1% (v/v) of ECGS and 10% (v/v) of heparin, or osteogenic medium, consisting of basal medium supplemented with β-glycerophosphate disodium salt (10 mM, Sigma-Aldrich), dexamethasone (10 nM, ThermoFisher Scientific) and ascorbic acid (50 μg.mL ^-1^, Sigma-Aldrich). Four conditions were prepared, namely capsules containing spheres and cultured in basal medium (termed as spheres basal), microdiscs in basal medium (termed as microdiscs basal), spheres in osteogenic medium (termed as spheres osteo) and microdiscs in osteogenic medium (termed as microdiscs osteo). The four types of capsules were cultured in triplicate in non-adherent 24-well plates at a density of 4 capsules per well. Each culture had its medium change twice a week (50% of the total volume).

### MTS viability assay

Capsules were tested for cytotoxicity and suitability for live cell encapsulation using a formazan-based colorimetric assay (CellTiter 96® AQueous One Solution Cell Proliferation Assay, Promega) at days 7, 14, and 21 of culture. Briefly, four capsules of each condition (spheres basal, microdiscs basal, spheres osteo, and microdiscs osteo) were placed in 1.5 mL centrifuge tubes with 400 μl of a MTS solution diluted in DPBS at a 1:6 ratio. Samples were incubated for 3 h at 37 °C and 5% of CO _2_, protected from light. After the incubation period, the capsules’ membrane was disrupted, and the released contents were centrifuged for 5 min at 400 g. 100 μl of the resultant supernatant of each condition was transferred in triplicate to a transparent 96-well plate. The amount of formazan product was measured by absorbance at a wavelength of 490 nm using a microplate multimode reader (Gen 5 2.01, Synergy HTX, Bio-TEK). MTS activity results were normalized with DNA quantification data.

### DNA and alkaline phosphatase activity quantification

After cell lysis, capsules were analyzed at days 3, 7, 14, and 21 of culture for DNA quantification and at days 7, 14, and 21 for alkaline phosphatase activity. DNA quantification and ALP activity were performed to assess the capsules’ ability to support cell proliferation and osteogenic differentiation, respectively. Briefly, four capsules of each condition (spheres basal, microdiscs basal, spheres osteo and microdiscs osteo) were collected in 1.5 mL centrifuge tubes and washed in DPBS. Samples were incubated with 1x trypsin-EDTA for 5 min at 37 °C and 5% of CO _2_. Cell aggregates were dissociated and washed again in DPBS. Samples were resuspended in 2% (v/v) of triton (Triton X-100 BioXtra, Sigma-Aldrich) in ultra-pure water, and placed in a shaking water bath at 37 °C for 30 min. Ultimately, samples were immediately stored at -20 °C until analysis. After defrosting, samples were used according to the kit recommendations (Quant-iTTM PicoGreen® dsDNA assay kit, Thermo Fisher Scientific). A standard curve was obtained with the provided dsDNA solution. After transferring each solution to a 96-well white opaque plate (in duplicate), the plate was incubated for 10 min at RT in the dark. Fluorescence was read at excitation of 485/20 nm and emission of 528/20 nm using a microplate multimode reader (Gen 5 2.01, Synergy HTX, Bio-TEK). ALP activity assay was performed with the remaining lysis solutions. A standard curve was obtained with a diluted series of 4-nitrophenol solution (10 mM, Sigma-Aldrich). Briefly, a substrate solution (pH 9.8) was prepared by dissolving 4-nitrophenylphosphate disodium salt hexahydrate (0.2% w/v, Sigma-Aldrich) in diethanolamine (1 M, Sigma-Aldrich). Each sample (25 μL) was mixed with the prepared substrate solution (75 μL). After 45 min at 37 °C in the dark, absorbance was read at 405 nm using a microplate multimode reader (Gen 5 2.01, Synergy HTX, Bio-TEK). ALP activity results were normalized with DNA quantification data.

### Osteopontin immunofluorescence staining

Capsules were collected in 1.5 mL centrifuge tubes and washed in DPBS. The capsules’ membrane was disrupted, and the released contents were fixed in a 4% (v/v) formaldehyde solution for 15 min at RT. After washing in PBS, samples were permeabilized with a 0.2% (v/v) triton-X solution (Triton X-100 BioXtra, Sigma-Aldrich) prepared in WFI for 5 min at RT. Non-specific binding was blocked using a solution of 5% (v/v) FBS prepared in PBS for 30 min at RT. Afterward, the samples were incubated with the primary antibody rabbit anti-human osteopontin (1:100 in 5% (v/v) FBS/PBS, 1 mg.mL ^-1^, BioLegend) overnight at 4 °C in an orbital shaker. After washing in PBS, samples were incubated for 1 h at RT with the secondary antibody chicken anti-rabbit AlexaFluor 647 (1:500 in 5% (v/v) FBS/PBS, 1 mg.mL ^-1^, BioLegend). Ultimately, samples were counterstained with DAPI (1:1000 diluted in PBS, 1 mg.mL ^-1^, Thermo Fisher Scientific) for 5 min at RT, and washed in PBS. Samples were visualized by fluorescence microscopy (Axio Imager 2, Zeiss).

### Scanning electron microscopy visualization

The surface of the produced spheres and microdiscs was visualized by scanning electron microscopy (SEM) to measure the length of such microparticles, and to confirm the presence of a successful nanogrooved pattern on microdiscs. Microparticles were gold palladium-sputtered (Polaron SEM Coating Unit E5000) and visualized using a Hitachi S4100 operating at 15.0 kV. Additionally, the contents of capsules, i.e. cells and microparticles, were also visualized after 7 and 21 days of culture. Capsules were fixed in 4% (v/v) formaldehyde for 15 min at RT, and then dehydrated using sequential ethanol dilutions, namely 40% (overnight), 50%, 60%, 70%, 80%, 90%, and 100% (v/v), 15 min each. Afterwords, capsules were disrupted by mechanical force to expose the inner content (cells and microparticles). Samples were gold palladium-sputtered (Polaron SEM Coating Unit E5000) and visualized using a Hitachi, SU-70 operating at 4 kV.

### Statistical analysis

All data were statistically analyzed using two-way analysis of variance (ANOVA) using the Tukey´s with multiple comparison tests. All results were expressed in the form of mean ± standard deviation. Analysis and the corresponding graphical representations were performed using GraphPad Prism 9.00 (An alternative software that can perform an equivalent analysis is Microsoft Excel). A *p*-value < 0.05 was considered statistically significant.

The total of the qualitative data (images, schemes, and graphics) and quantitative data of this research paper are available for download and consultation ^68^.

## Results

### Cell isolation and characterization

**Figure 2.**
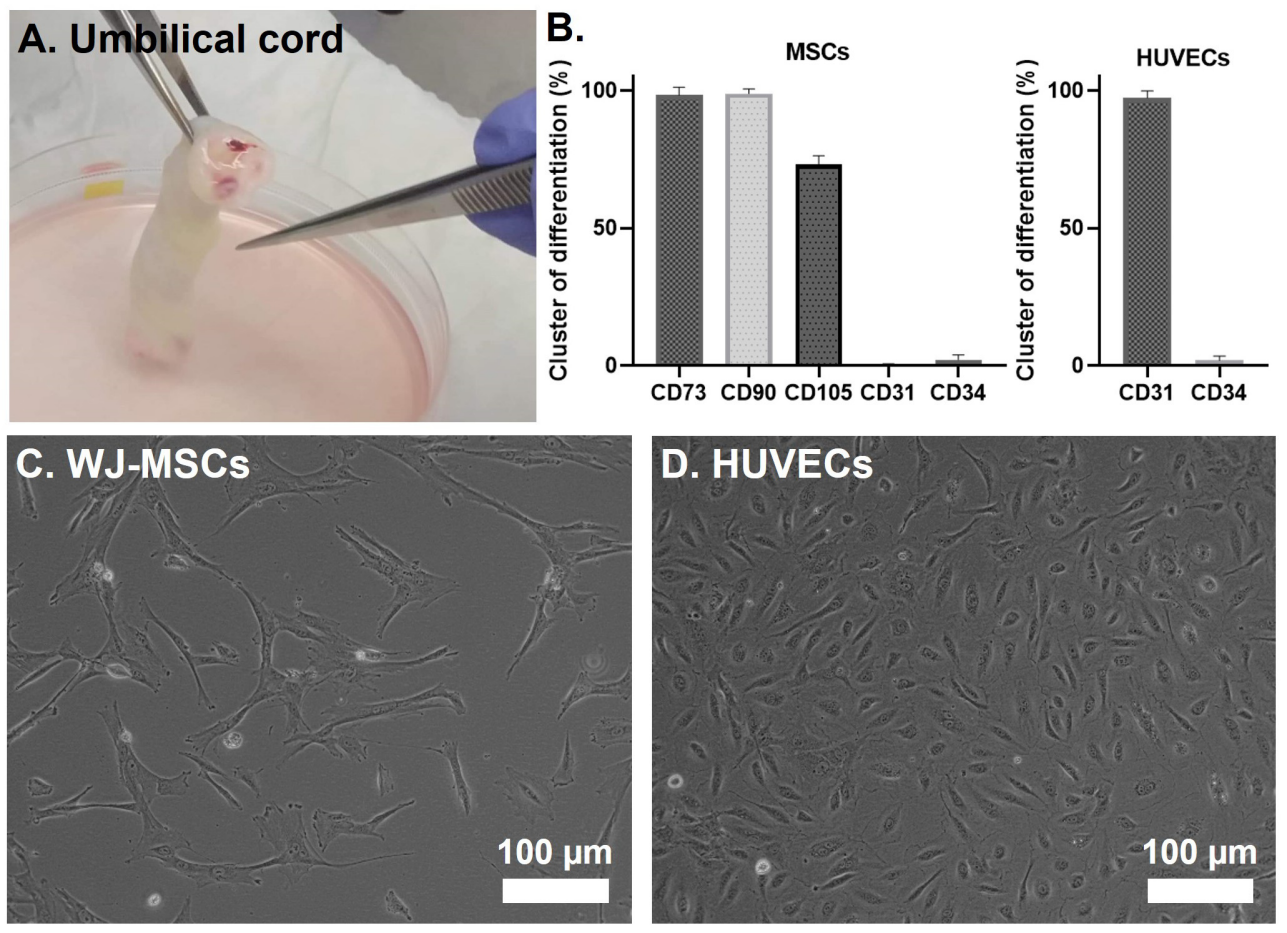
( **A**) Representative image of an UC piece. ( **B**) Flow cytometry analysis of WJ-MSCs and HUVECs after isolation. Cells were analyzed in passages 2 and 3, respectively. ( **C**– **D**) Inverted light microscope images of a 2D culture of WJ-MSCs and HUVECs, respectively.

After isolation of the WJ-MSCs and HUVECs from two separate human UC ( Figure 2A), cells were expanded on adherent culture flasks. The successful isolation of WJ-MSCs and HUVECs was further confirmed by flow cytometry ( Figure 2B). In the case of WJ-MSCs, an evident expression of the mesenchymal stem cell markers CD90, CD105, and CD73 was detected, while the hematopoietic marker CD34 and the endothelial marker CD31 were notably absent. CD31 was particularly chosen as a negative stemness marker due to the spatial proximity of the WJ to the human umbilical vein. In the case of the HUVECs, they were confirmed by the positive expression of CD31, a marker universally present in all endothelial cell types ^69^. The hematopoietic marker CD34 exhibited minimal expression levels (≤ ~ 3%) in HUVECs, thus being considered absent. Although CD34 is occasionally employed as an endothelial marker, it is mainly associated with early endothelial progenitor cells rather than fully mature endothelial cells, as HUVECS ^70, 71^. After observing the phenotype of isolated cells by light microscopy, WJ-MSCs displayed a fibroblast-like morphology ( Figure 2C), and HUVECs a cobblestone shape ( Figure 2D) which corresponds to its already reported morphology ^71, 72^. Following the successful isolation of the required cell types, WJ-MSCs and HUVECs were independently expanded in culture until reaching the desired cell density for subsequent encapsulation. Since HUVECs are suggested to show a more prominent culture medium-dependence *in vitro* than MSCs, the endothelial M199 medium was chosen as the basal medium for the *in vitro* capsules culture ^70^. For this reason, before the encapsulation procedure, WJ-MSCs were assessed for viability in the endothelial medium and cells displayed a fibroblast-like morphology, similarly to the control with α-MEM (data not shown).

### Microparticles’ characterization

**Figure 3.**
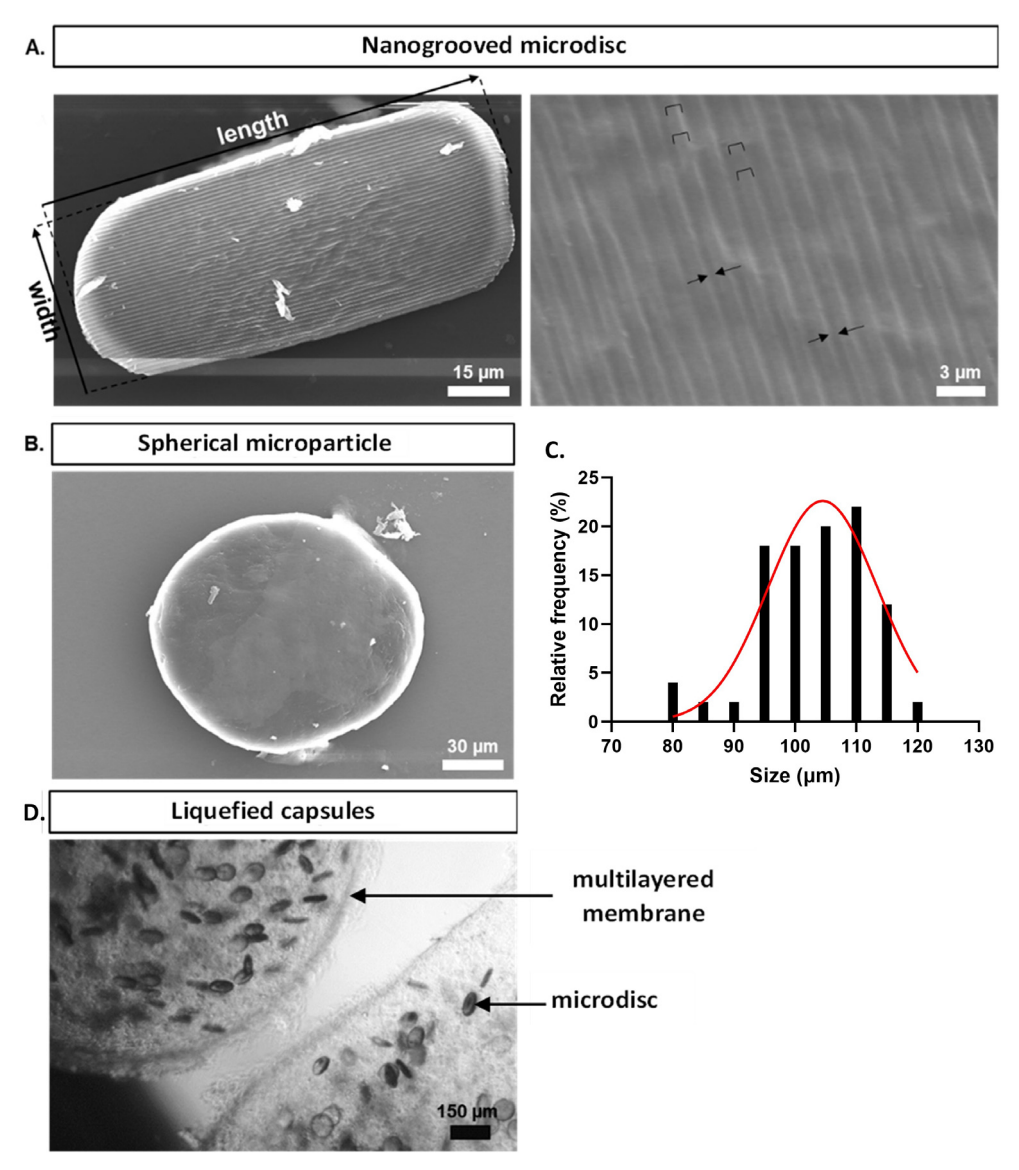
( **A**) Scanning electronic microscopy representative images of the nanogrooved microdisc and detailed nanogrooved topography. Ridges are filled with staples and grooves are delimited by arrows. ( **B**) Scanning electronic microscopy representative image of a spherical microparticle within 80 to 100 µm. ( **C**) Size distribution (diameter) of spherical microparticles within 80 to 100 µm. Diameter measurements were performed using Image J software (n=50). ( **D**) Representative inverted light microscope image of a liquefied multilayered capsule laden with WJ-MSCs, HUVECs, and microdiscs at day 0.

After production, polycaprolactone microparticles were plasma treated, coated with collagen type I, and observed by SEM. The microdiscs exhibited an average length of 103 ± 25 μm and width of 60 ± 18 μm, where the presence of the nanogrooved micropatterning was clearly observed ( Figure 3A). The microdiscs displayed grooves with dimensions of 358 ± 23 nm, spaced apart by ridges measuring 944 ± 49 nm, and possessing a height of 201 ± 45 nm. The CDs utilized to imprint the pattern on the PVA counter-molds had groove widths of 412 ± 12 nm, ridge widths of 1185 ± 16 nm, and ridge heights of 197 ± 14 nm (data not shown). The control group was constituted by spherical microparticles sieved between 80 and 100 µm, as shown in Figure 3B. The diameter of the control spherical microparticles was measured in the Image J software and their size distribution is shown in Figure 3C. The size distribution of spherical microparticles within 25 to 40 µm is already described in a previous work ^62^.

### Bioencapsulation and cell viability

**Figure 4.**
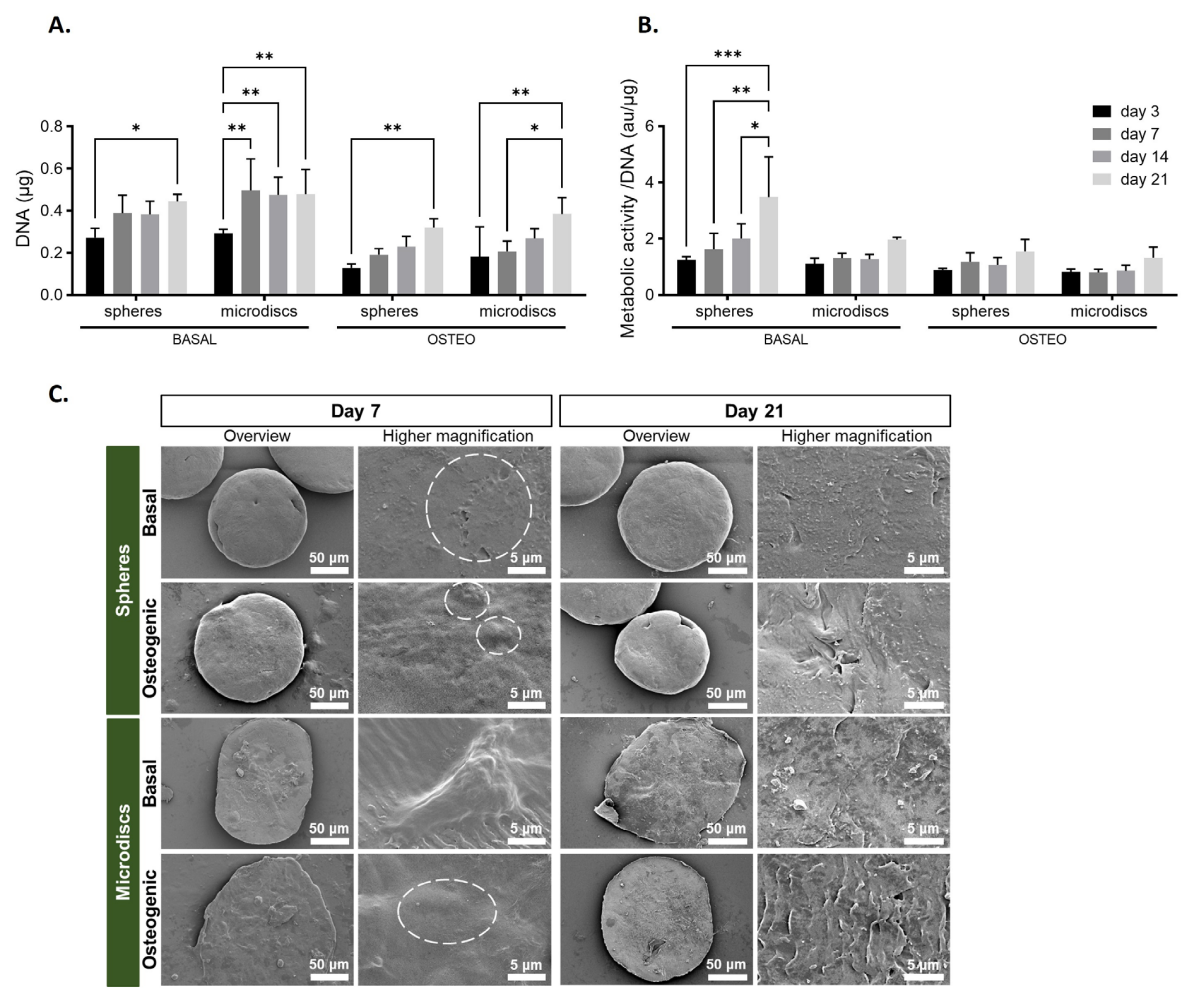
( **A**) DNA quantification assay of capsules with spherical (spheres) or nanogrooved microparticles (microdiscs) cultured in basal or osteogenic medium for 21 days. ( **B**) Cell metabolic activity assay determined by MTS colorimetric assay and normalized by DNA. *p*-values < 0.05 were considered statistically significant. (****p < 0.0001; ***p < 0.001; **p < 0.01; *p < 0.05). ( **C**) Scanning electron microscopy (SEM) of capsules at days 7 and 21 post-encapsulation (white dotted circumferences are outlining the cells).

Multilayered capsules were generated containing the co-culture system (WJ-MSCs and HUVECs), and microdiscs or spheres acting as cell adhesion sites within the liquefied core ( Figure 3C). All the capsules remained stable during the 21 days of culture, without cells or microparticles escaping. The total DNA content was quantified on days 3, 7, 14, and 21 to provide an indirect assessment of cell number over time ( Figure 4A). For all conditions, cell number increased significantly from day 3 to day 21. When comparing DNA quantity of co-encapsulated cells with microdiscs, with their control (spheres) cultured in the same medium, no significant differences were found at day 3 and 21. In basal medium, a tendency for increased cell growth was observed from day 3 to day 7 (statistically significant only in microdiscs), with growth remaining stable until day 21. In contrast, cells in osteogenic medium showed a gradual increase in cell growth from day 3 to day 21.

The cell metabolic activity of co-cultured WJ-MSCs and HUVECs was evaluated by MTS colorimetric assay at days 3, 7, 14, and 21 days of culture and normalized by the DNA content ( Figure 4B). In the basal medium, cells co-cultured with spheres exhibited a gradual and significant increase in metabolic activity from day 3 to day 21. In the other conditions, cells maintained a constant metabolic activity from day 3 to day 14, followed by a slight increase up to day 21 (not statistically significant).

The visualization of the inner content of capsules by SEM ( Figure 4C) revealed the deposition of ECM covering the total surface of microparticles on the last day of culture. On day 7, cells on the surface of microparticles appeared as protuberances (outlined by dotted circumferences), since they were not fully embedded within the newly deposited ECM. Higher magnification of microdiscs in basal medium highlighted a cell adhered to the nanogroove pattern, showing that the surface of the microdiscs is still not completely covered by the newly deposited ECM.

### Osteogenic differentiation

**Figure 5.**
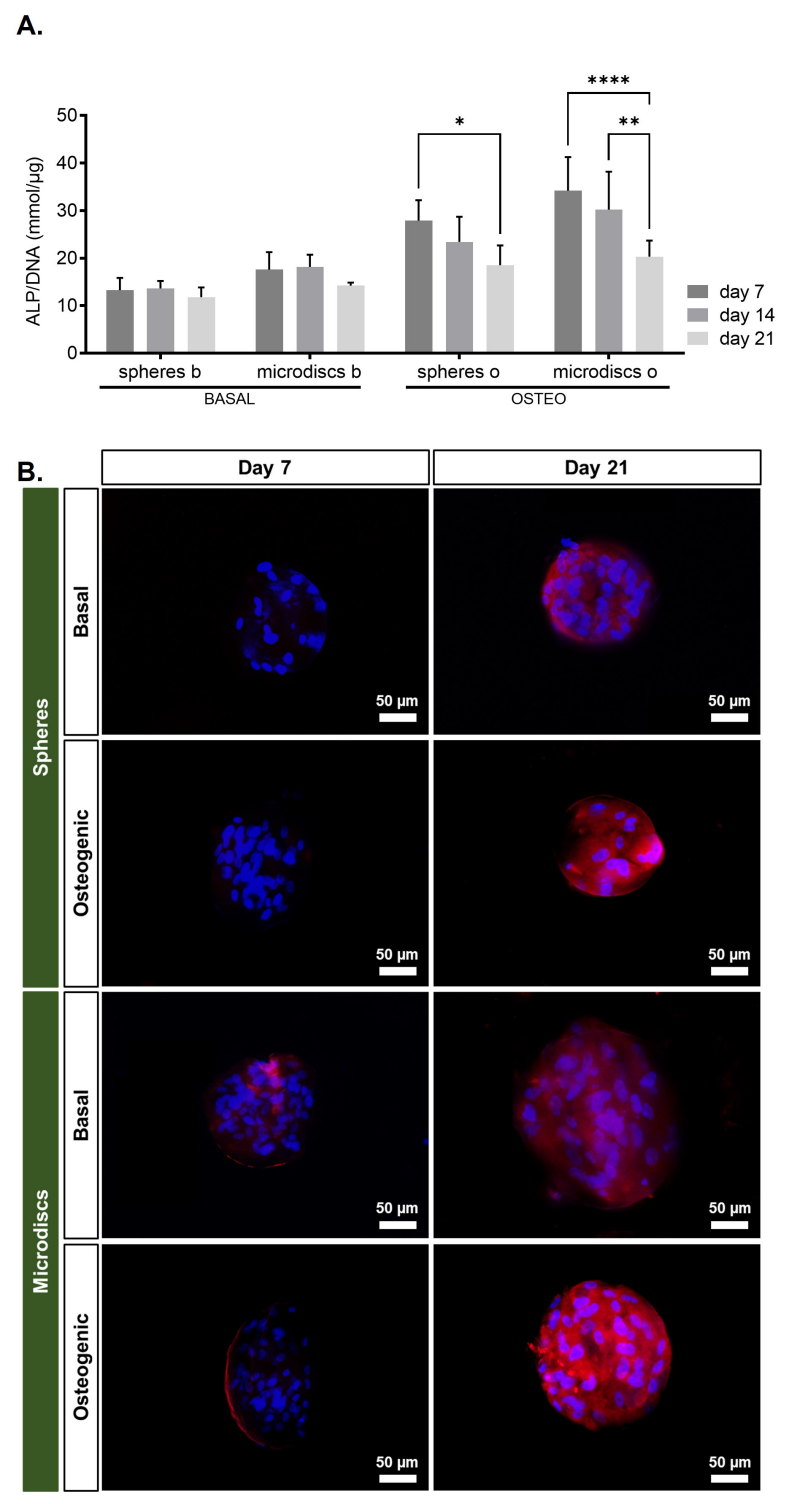
( **A**) ALP quantification assay of capsules with spherical microparticles (spheres) or nanogrooved microdiscs (microdiscs) in basal medium or osteogenic medium at 7, 14, and 21 days of culture. *p*-values < 0.05 were considered statistically significant. (****p < 0.0001; ***p < 0.001; **p < 0.01; *p < 0.05). ( **B**) Assessment of osteogenic markers by fluorescence microscopy, osteopontin and DAPI at days 7 and 21 post-encapsulation.

Osteogenic differentiation commitment was assessed by ALP activity quantification on days 7, 14, and 21 of culture ( Figure 5A) and by osteopontin detection on days 7 and 21 by fluorescence microscopy ( Figure 5B). The ALP results were normalized per DNA content.

In spheres and microdiscs cultured in osteogenic medium, ALP activity achieved a peak at day 7, but progressively decreased significantly until day 21. Comparatively, ALP activity was lower in spheres and microdiscs cultured in basal medium and did not change over time.

These results showed that osteoblastic differentiation was significantly higher in osteogenic medium, especially at early time points, for both spheres and microdiscs.

The immunofluorescence images of the capsules on days 7 and 21 indicated that cells were able to adhere to the surface of both spheres and microdiscs, evidenced by the DAPI nucleus staining surrounding the microparticles. On day 7, osteopontin fluorescence could not be detected in basal or osteogenic spheres. However, osteopontin was expressed in spheres cultured in both media on day 21. In opposition, in microdiscs, osteopontin could be visualized at days 7 and 21, when cultured in basal or osteogenic medium.

## Discussion

Herein, we present a 3D bioencapsulation system composed of liquefied and multilayered capsules that contain three essential elements, namely (1) surface functionalized microparticles, which provide adhesion sites for cells, and can also act as bioinstructive materials to aid WJ-MSCs differentiation; (2) cells, which are freely dispersed within the liquefied core microenvironment and can self-organize into a 3D culture according to their specific needs; and (3) a permselective multilayered membrane that wraps the liquefied core of capsules, ensuring permeability to essential molecules for cell survival while avoiding the entry of larger molecules and cells from the immunological system. Nevertheless, it is expected a minimal and controlled immune reaction due to the biotolerability of the materials employed ^73^. Additionally, the membrane confers flexibility to the capsule, maintaining its integrity during implantation by injection, and maximizing the direct contact between the core contents (e.g. cell-cell and cell-microparticle interactions). This disruptive bioencapsulation system has already been tested *in vitro* and *in vivo* ^62, 63, 65^ however, this is the first time that the system incorporates nanogrooved microdiscs and cells sourced just from the UC tissue, conventionally considered as biological waste.

As previously referred, the present study aimed to assess the potential of nanogrooved microdiscs in the osteogenic differentiation of WJ-MSCs co-cultured with HUVECs, without adding supplemental osteogenic factors to the culture medium. Considering that MSCs can sense topographical features (mechanotransduction) and differentiate towards the osteoblastic lineage ^51– 53, 74– 77^, we examined the inherent potential of nanogrooved microdiscs as facilitators of osteoblastic differentiation in a basal medium.

As expected, cells adhered to both spheres and microdiscs showing an *in vitro* proliferation potential, visualized by the immunofluorescence and SEM images. These results corroborate the suitability of the system to support WJ-MSCs and HUVECs metabolism and proliferation during the following *in vitro* culture tests.

Bone formation is progressively achieved by employing three fundamental stages: i) proliferation, ii) extracellular matrix production and maturation, and iii) mineralization ^78, 79^.

Normally, under osteogenic differentiation, proliferation characteristically occurs first, increasing the cell number and only after is observed the osteoblastic differentiation, observed by a peak of ALP activity. Around day 7 to day 14 osteoblastic differentiation is prominent represented by an increase in ALP activity until day 14, and osteoblasts actively produce the bone ECM. Then, the ALP activity decreases after day 14 until day 21, which correspond to matrix mineralization. The late osteogenic marker osteopontin is reported to present *in vitro* a peak expression during the mineralization phase, from day 14 to 21 ^80^. In fact, the ALP activity results of capsules in osteogenic medium evidenced an early osteoblastic differentiation at day 7, following a progressive decline until day 2, which might be associated with matrix mineralization. However, the corresponding DNA quantification showed a progressive increase in cell number from day 3 to day 21. This might indicate that under osteogenic differentiation, encapsulated WJ-MSCs started to differentiate early towards osteoblasts, which proliferated until the last day of culture. Comparing ALP activity between spheres and microdiscs cultured in osteogenic and basal medium, it was found a higher osteoblastic differentiation in microdiscs. This can indicate that microdiscs contribute to WJ-MSCs differentiation towards the osteogenic lineage, which suggests nanogrooved microdiscs would be able to induce osteoblastic differentiation even in the absence of supplemental osteogenic differentiation factors.

Regarding osteopontin secretion, this late osteogenic marker is usually expressed during the matrix mineralization phase, with a peak between days 14 and 21 ^81^. It is also known that β-glycerophosphate is required as a source of phosphate in the mineralization of the ECM. Since β-glycerophosphate was present in the osteogenic medium, the mineralization of the ECM was chemically induced. Remarkably, although β-glycerophosphate was not present in the basal medium, osteopontin was already detected by the immunofluorescent assay on day 7 for cells cultured with microdiscs. It was already expected that matrix mineralization would occur in microdiscs basal since a previous work using nanogrooved microdiscs as osteogenic differentiation vehicles reported that finding ^62^.

## Conclusion and future directions

This work shows the osteoblastic differentiation of WJ-MSCs driven by nanogrooved microdiscs in a 3D system, without requiring the addition of supplemental osteogenic differentiation factors. This system represents a great advance in the field of bone tissue regeneration, permitting the construction of a bone scaffold with minimum *in vitro* manipulation. However, it still needs further investigation to assess its performance in bone tissue vascularization, and its potential to establish vascular networks within the host vasculature, once implanted. The use of WJ-MSCs and HUVECs isolated from the same UC tissue is also an important step to investigate in the future, that could open the possibility to explore the field of personalized medicine.

## Ethics and consent

The collected tissues were obtained under a cooperation agreement between the Aveiro Institute of Materials - University of Aveiro and Hospital do Baixo Vouga (Aveiro, Portugal) after approval of the Competent Ethics Committee (CEC) dated 20 ^th^ October 2020. The received human tissues were handled under the guidelines approved by the CEC. Written informed consent was obtained from all subjects.

## Data Availability

Zenodo: Underlying data for ‘Liquefied capsules containing nanogrooved microdiscs and umbilical cord-derived cells for bone tissue engineering’, https://doi.org/10.5281/zenodo.13627199 ^68^. This project contains the following underlying data organized by the research paper figures: The total of the qualitative data (images, schemes and graphics) displayed in the research paper. The total of the quantitative data comprised in GraphPad files, as well as in an open-source program (Microsoft Excel) as an alternative. Data are available under the terms of the Creative Commons Attribution 4.0 International license (CC-BY 4.0)
