# Supplementary material for: Liquefied capsules containing nanogrooved microdiscs and umbilical cord-derived cells for bone tissue engineering
Source: Open Res Eur. 2024 Sep 9;4:94. Originally published 2024 Apr 25. [Version 2] doi: 10.12688/openreseurope.17000.2 (PMC11393531; doi:10.12688/openreseurope.17000.2)
Supplement: Peer review history

# Reviewer report: Reviewer response for version 2

**DOI:** 10.21956/openreseurope.20008.r44071

**Published:** 2024-09-19

**Authors:** Jeroen Rouwkema

**Affiliations:** 1 University of Twente Faculty of Engineering Technology, Enschede, The Netherlands

The authors have carefully addressed the reviewer comments which improved the manuscript quality. I approve of the current manuscript.

Is the study design appropriate and does the work have academic merit?

Partly

Is the work clearly and accurately presented and does it cite the current literature?

Yes

If applicable, is the statistical analysis and its interpretation appropriate?

Yes

Are all the source data underlying the results available to ensure full reproducibility?

Yes

Are the conclusions drawn adequately supported by the results?

Partly

Are sufficient details of methods and analysis provided to allow replication by others?

Yes

Reviewer Expertise:

tissue engineering, biofabrication, mechanobiology

I confirm that I have read this submission and believe that I have an appropriate level of expertise to confirm that it is of an acceptable scientific standard.

# Reviewer report: Reviewer response for version 2

**DOI:** 10.21956/openreseurope.20008.r44069

**Published:** 2024-09-12

**Authors:** Paula Vazquez

**Affiliations:** 1 Centro de Investigación Cooperativa en Biomateriales, San Sebastián, Spain

The authors have answered all the questions and provided complementary references as well as data.

Is the study design appropriate and does the work have academic merit?

Yes

Is the work clearly and accurately presented and does it cite the current literature?

Yes

If applicable, is the statistical analysis and its interpretation appropriate?

Yes

Are all the source data underlying the results available to ensure full reproducibility?

Partly

Are the conclusions drawn adequately supported by the results?

Partly

Are sufficient details of methods and analysis provided to allow replication by others?

Yes

Reviewer Expertise:

3D in vitro cancer models

I confirm that I have read this submission and believe that I have an appropriate level of expertise to confirm that it is of an acceptable scientific standard.

# Reviewer report: Reviewer response for version 1

**DOI:** 10.21956/openreseurope.18371.r40874

**Published:** 2024-08-14

**Authors:** Mani Diba, Yannick Hajee, Farhad Sanaei

**Affiliations:** 1 Radboud University Medical Center, Nijmegen, The Netherlands

This manuscript reports the use of a 3D bioencapsulation system for co-culture of mesenchymal stem cells and endothelial cells derived from the human umbilical cord. The authors investigate the encapsulation of cells along with microspheres or nanogrooved microdiscs in capsules made of a semipermeable membrane containing a liquefied alginate core. The study specifically focuses on the osteogenic differentiation of cells in this system using both basal and osteogenic media and the impact of microspheres versus nanogrooved microdiscs. Characterizations have been carried out by means of a range of biochemical and microscopic methods, which suggest that topological features, when employing the capsules containing nanogrooved microdiscs, might facilitate the osteogenic differentiation of stem cells. The reported approach is interesting and presents a novel methodology for bone tissue engineering. However, the manuscript would benefit from the following revisions.

**COMMENTS:**

1. The different morphology of disks with nanogrooves compared to spherical particles could conceivably influence the outcome of the experiment either directly (e.g., effect of surface curvature on cells) or indirectly (e.g., if particles sediment in the liquefied core, then microdiscs may result in different packing than microspheres). The authors are advised to discuss whether such differences could have influenced the outcome of the experiments.
2. It is not clear whether the microparticles in the liquefied environment within the capsules remain dispersed or aggregated/sedimented. The authors are advised to elaborate on this point in the manuscript.
3. The authors should clarify if WJ-MSCs and HUVECs were isolated from the same or different umbilical cords. If they were from different cords, the authors are advised to elaborate on why both cell types were not isolated from the same donor, given that using both cell types from a single source is mentioned as an advantage in the manuscript.
4. It is not clear whether the presence of HUVECs is necessary in this study or whether MSC-only cultures may exhibit increased osteogenic differentiation due to surface topography as well. The authors are advised to clarify this point in the discussion section.
5. The authors should specify in the Figure 5 caption which color corresponds to DAPI and which to osteopontin.
6. The authors have employed ALP activity and osteopontin as markers for osteogenic differentiation. As described in the discussion section, mineralization is another key marker of bone formation. The authors state, “It was already expected that matrix mineralization would occur in microdiscs,” but it is unclear whether mineralization was observed within the timeframe of the experiment. Elaboration on this point would strengthen the manuscript.

**Suggestions for Minor Language Revisions:**

- Page 4 paragraph 2 (p4p2): “Late” strategy should be “latest”.
- P4p2: like perivascular “cells”.
- P6p5: “were added dropwise” instead of “were dropwised”.
- P11p1: day 7 instead of day 1.

Is the study design appropriate and does the work have academic merit?

Partly

Is the work clearly and accurately presented and does it cite the current literature?

Yes

If applicable, is the statistical analysis and its interpretation appropriate?

Yes

Are all the source data underlying the results available to ensure full reproducibility?

Yes

Are the conclusions drawn adequately supported by the results?

Partly

Are sufficient details of methods and analysis provided to allow replication by others?

Yes

Reviewer Expertise:

Biomaterials, Tissue Engineering, Bone Regeneration

We confirm that we have read this submission and believe that we have an appropriate level of expertise to confirm that it is of an acceptable scientific standard, however we have significant reservations, as outlined above.

# Reviewer report: Reviewer response for version 1

**DOI:** 10.21956/openreseurope.18371.r40876

**Published:** 2024-08-14

**Authors:** Tiziano Serra

**Affiliations:** 1 AO Research Institute, Clavadelerstrasse, Davos Platz, Switzerland

The manuscript describes a study on the role of surface topography in the differentiation of Wharton's jelly-derived mesenchymal stem cells and human umbilical vein endothelial cells. Cells were encapsulated in liquefied capsules containing either spherical microparticles or nanogrooved microdiscs, maintaining high viability throughout the culture period. Results indicated that nanogrooved microdiscs significantly enhanced osteogenic differentiation, even without osteogenic medium. This suggests the potential of using topographical features for bone tissue engineering. 

 The manuscript is clear and well written. However, few points could be addressed to improve it:

- Does the presence of microdiscs or microspheres affect the shape retention of liquefied capsules  during culturing? Is there any degradation of the capsules along time and or diffusion of media?
- In Figure 5B, it is not clear if the OPN staining is related to the microsphere loaded with capsules or with nanogrooved microdiscs. This makes unsure the final claim and conclusion of the manuscript.

Is the study design appropriate and does the work have academic merit?

Yes

Is the work clearly and accurately presented and does it cite the current literature?

Yes

If applicable, is the statistical analysis and its interpretation appropriate?

Yes

Are all the source data underlying the results available to ensure full reproducibility?

Yes

Are the conclusions drawn adequately supported by the results?

Partly

Are sufficient details of methods and analysis provided to allow replication by others?

Yes

Reviewer Expertise:

biofabrication, biomaterials, tissue engineering

I confirm that I have read this submission and believe that I have an appropriate level of expertise to confirm that it is of an acceptable scientific standard, however I have significant reservations, as outlined above.

# Reviewer report: Reviewer response for version 1

**DOI:** 10.21956/openreseurope.18371.r40875

**Published:** 2024-06-07

**Authors:** Jeroen Rouwkema

**Affiliations:** 1 University of Twente Faculty of Engineering Technology, Enschede, The Netherlands

This manuscript by Carreira et al describes an interesting study on the effect of nanogrooved microdiscs on the osteogenic differentiation of umbilical cord-derived cocultures of endothelial cells and mesenchymal stromal cells in a liquefied capsule environment. The manuscript shows that cells cultured in the presence of nanogrooved microdiscs are able to proliferate and display osteogenic differentiation as evidenced by the expression al alkaline phosphatase and osteopontin. It also shows that osteogenic differentiation appears to be increased when comparing with the same cells that are cultured in the presence of non-patterned spherical microparticles.

 The manuscript is well written and overall sound. However, several points could be addressed to improve the manuscript:

- Overall, the study compares cell cultures in the presence of flat microdiscs that contain a nanogrooved surface pattern with cultures in the presence of spherical microparticles that do not contain a surface pattern. This choice of the control group (spherical particles) is not optimal, as it does not isolate a single characteristic which can then be linked with observed cell behaviour. With the current setup, observed difference could for instance be due to the presence or absence of the surface patterns, but could also be due to using a microdisc versus using a microsphere, which has a clear curvature compared to the flat disc. A better control would had been using microdiscs without the nanogrooved pattern. Even though the manuscript does not claim that the observed effects are due to the surface pattern, the choice of the control group makes it difficult to draw conclusions regarding the value of the patterned discs.
- Please carefully check the numbering of the references. On page 4, right  column, third paragraph for instance, it looks like the references referring to ‘differentiation through contact guidance’ should not start at 33, but somewhere around 40 – 42.
- Page 9 results regarding cell viability: the DNA quantification over time shows that the number of cells increases over time. It does not show that the proliferation (the rate of increase of nr of cells) increases over time. To make this conclusion, more time points and for instance an analysis of the doubling time over time would had been needed
- Page 10 first paragraph regarding cell metabolic activity: please indicated whether the values are corrected for the number of cells (DNA content). If not, the metabolic activity per cell seems to decrease, as the number of cells increases over the same timeframe.
- Page 12 right paragraph line 2-5: according to the presented results ALP does not increase between days 7 to 14. If anything, there seems to be a downwards trend in the osteogenic medium.   

Is the study design appropriate and does the work have academic merit?

Partly

Is the work clearly and accurately presented and does it cite the current literature?

Yes

If applicable, is the statistical analysis and its interpretation appropriate?

Yes

Are all the source data underlying the results available to ensure full reproducibility?

Yes

Are the conclusions drawn adequately supported by the results?

Partly

Are sufficient details of methods and analysis provided to allow replication by others?

Yes

Reviewer Expertise:

tissue engineering, biofabrication, mechanobiology

I confirm that I have read this submission and believe that I have an appropriate level of expertise to confirm that it is of an acceptable scientific standard, however I have significant reservations, as outlined above.

# Response

**Published:** 2024-09-02

**Authors:** João Mano

**Affiliations:** Chemistry Department - CICECO, University of Aveiro, Aveiro, Aveiro District, Portugal

**C1. Overall, the study compares cell cultures in the presence of flat microdiscs that contain a nanogrooved surface pattern with cultures in the presence of spherical microparticles that do not contain a surface pattern. This choice of the control group (spherical particles) is not optimal, as it does not isolate a single characteristic which can then be linked with observed cell behaviour. With the current setup, observed difference could for instance be due to the presence or absence of the surface patterns, but could also be due to using a microdisc versus using a microsphere, which has a clear curvature compared to the flat disc. A better control would had been using microdiscs without the nanogrooved pattern. Even though the manuscript does not claim that the observed effects are due to the surface pattern, the choice of the control group makes it difficult to draw conclusions regarding the value of the patterned discs.**

 R1. We acknowledge the reviewer for this thoughtful comment regarding the choice of the control group. It is already described in a previous paper from the group that spherical microparticles closer to the upper diameter range (including 80 µm) can exhibit a more flattened and less spherical shape, which is likely to happen due to collapse during production (Bjørge et al., ACS Appl Mater Interfaces., 2022, 4;14(17):19116-19128). The paper also demonstrates that in a co-culture of mesenchymal stem cells and HUVECs, the expression of hydroxyapatite (an osteogenic marker) after 21 days of culture with flat, non-patterned microdiscs, follows a similar trend to that observed with microspheres, for both basal and osteogenic media. For these reasons, we consider that these spherical microparticles serve as suitable controls for the primary variable being tested in this study, which is nanogrooved topography.

**C2. Please carefully check the numbering of the references. On page 4, right  column, third paragraph for instance, it looks like the references referring to ‘differentiation through contact guidance’ should not start at 33, but somewhere around 40 – 42.**

 R2. Thank you for your scrutinous revision regarding the references. We have already checked all the references and made all the necessary changes.  

**C3. Page 9 results regarding cell viability: the DNA quantification over time shows that the number of cells increases over time. It does not show that the proliferation (the rate of increase of nr of cells) increases over time. To make this conclusion, more time points and for instance, an analysis of the doubling time over time would have been needed.**

 R3. We thank the reviewer for this comment. The DNA content is correlated directly with cell division, and since it is increased the number of cells is increasing as well. However, we do not have the required data to affirm that the proliferation increases over time. Hence, to be more scientifically accurate we have made all the necessary changes to the manuscript according to this comment.

**C4. Page 10 first paragraph regarding cell metabolic activity: please indicate whether the values are corrected for the number of cells (DNA content). If not, the metabolic activity per cell seems to decrease, as the number of cells increases over the same timeframe.**

 R4. We acknowledge the reviewer's comment regarding the normalization of the metabolic activity tests. The results initially presented were not corrected for DNA content. In response, we have revised the manuscript and replaced the original cell metabolic activity results with those normalized by DNA content, as shown in **Figure 4B**.

**C5. Page 12 right paragraph lines 2-5: according to the presented results ALP does not increase between days 7 to 14. If anything, there seems to be a downward trend in the osteogenic medium.**

 R5. We thank the reviewer attention for this mistake.  We have revised the manuscript and made the necessary corrections to maintain consistency and accordance.

# Reviewer report: Reviewer response for version 1

**DOI:** 10.21956/openreseurope.18371.r40500

**Published:** 2024-05-31

**Authors:** Paula Vazquez

**Affiliations:** 1 Centro de Investigación Cooperativa en Biomateriales, San Sebastián, Spain

**Summary**:

 The manuscript presents an innovative system that integrates a co-culture of human umbilical vein endothelial cells and Wharton’s jelly-derived mesenchymal stem cells for the study of osteogenic differentiation in 3D topographical context. This system employs a bioencapsulation technique involving a multilayered membrane encapsulating a liquefied core with nanogrooved microdiscs and the co-cultured cells.

**Strengths**:

 - The study's objectives and methodologies are clearly articulated.

 - The experimental design is robust, and the referencing is comprehensive.

**Potential improvements**:

 1. Donor Variability:

 The study would benefit from an increased number of umbilical cord donors to create a pool for both cell types, enhancing the robustness and generalizability of the findings.

 2. Cell Proportion and Representation:

 Have the authors considered evaluating different proportions of HUVECs and WJ-MSCs? It is crucial to determine whether the cell ratios used are representative of the tissues being modeled.

 3. Pre-Day 3 Assessment:

 How do the authors ensure that the spheres are correctly loaded before day 3 or any subsequent immunostaining or fluorimetric testing? Please clarify the assessment methods used.

 4. Membrane Permeability:

 Although the fabrication method has been previously described and cited, the diffusion characteristics of biomolecules through the multilayer membrane remain unclear. An experiment utilizing fluorescent probes of varying molecular weights could effectively demonstrate the membrane's permeability.

 5. Size Distribution of Spheres:

 Additional images showing the size distribution of the spheres are necessary to evaluate the reproducibility of the fabrication process.

 6. DNA Quantification Standards:

 The use of Lambda DNA as a standard for mammalian DNA quantification is suboptimal. The authors are advised to consider calf thymus DNA standards (ThermoFisher, Ref. 15633019) for more accurate quantification.

 7. Metabolic Activity Data (Figure 4B):

 There is an absence of metabolic activity data for day 3 in Figure 4B. The authors should provide an explanation for this omission.

 8. SEM Image Clarity (Figure 4C):

  The arrows indicating cells in the SEM images are difficult to discern. Profiling with a dotted line would enhance visibility. Additionally, have the authors examined the inner content of the capsules via SEM by sectioning them? The claim of higher ECM density is not convincingly supported by the SEM images provided. Further comments or additional images are required.

 9. Improving Immunostaining (Figure 5B):

 The use of additional blocking biomolecules (e.g., BSA or serum from the secondary antibody host) could potentially enhance the quality of immunostaining.

 10. Controls for Immunostaining (Figure 5B):

  Immunostaining images of empty capsules, spheres, and microdiscs should be included as controls. Moreover, immunostaining results for day 14 are missing and should be provided.

 11. Vascular Population Staining:

 Complementary immunostainings for vascular populations would confirm their presence, which is critical given that the co-culture system is a focal point of the study.

**Conclusion**:

 While the manuscript presents significant scientific contributions, it requires supplementary data and some textual revisions to enhance its relevance and impact. Addressing the aforementioned points will strengthen the validity and reliability of the study.

Is the study design appropriate and does the work have academic merit?

Yes

Is the work clearly and accurately presented and does it cite the current literature?

Yes

If applicable, is the statistical analysis and its interpretation appropriate?

Yes

Are all the source data underlying the results available to ensure full reproducibility?

Partly

Are the conclusions drawn adequately supported by the results?

Partly

Are sufficient details of methods and analysis provided to allow replication by others?

Yes

Reviewer Expertise:

3D in vitro cancer models

I confirm that I have read this submission and believe that I have an appropriate level of expertise to confirm that it is of an acceptable scientific standard, however I have significant reservations, as outlined above.

# Response

**Published:** 2024-09-02

**Authors:** João Mano

**Affiliations:** Chemistry Department - CICECO, University of Aveiro, Aveiro, Aveiro District, Portugal

**Comment 1.** Donor Variability: The study would benefit from an increased number of umbilical cord donors to create a pool for both cell types, enhancing the robustness and generalizability of the findings.

**Response 1.** We acknowledge the reviewer's comment. We completely agree that increasing the number of umbilical cord donors would increase the robustness of the findings. However, we did not have access to more umbilical cords at that time.

**Comment 2.** Cell Proportion and Representation: Have the authors considered evaluating different proportions of HUVECs and WJ-MSCs? It is crucial to determine whether the cell ratios used are representative of the tissues being modeled.

**Response 2.** Thank you very much for the interesting question. The ratio (1:1) of stromal and endothelial cells was established in our research group from previous studies, using the same system, liquified capsules (Correia et al., Adv Healthc Mater., 2019, 8, 1901221; Correia et al., Acta Biomater., 2017, 53: 483–494; Nadine et al., Adv Healthc Mater., 2022, 11, 2200651; Bjørge et al., ACS Appl Mater Interfaces., 2022, 4;14(17):19116-19128).

**Comment 3.** Pre-Day 3 Assessment: How do the authors ensure that the spheres are correctly loaded before day 3 or any subsequent immunostaining or fluorimetric testing? Please clarify the assessment methods used.

**Response 3.** Very interesting question. As is displayed in **Figure 3C** for the microdiscs, macrocapsules containing spheres were also visualized on the inverted light microscope upon production. This was a standard practice every time capsules were produced, either loaded with spheres or microdiscs.

**Comment 4.** Membrane Permeability: Although the fabrication method has been previously described and cited, the diffusion characteristics of biomolecules through the multilayer membrane remain unclear. An experiment utilizing fluorescent probes of varying molecular weights could effectively demonstrate the membrane's permeability.

**Response 4.** We acknowledge the relevance of the question about membrane permeability. The fabrication process of the multilayered membrane relies on the electrostatic interactions between oppositely charged polyelectrolytes, specifically poly(L-lysine), alginate, and chitosan. Our prior research, as documented in previous studies (Correia et al., Soft Matter., 2013, 9, 2125; Correia et al., Biomacromolecules, 2013, 14, 743), has already characterized the ability of the multilayered membrane to facilitate cell encapsulation using liquefied capsules. It has been demonstrated that the multilayered membrane permits the diffusion of crucial molecules necessary for cell survival. Additionally, it allows the diffusion of osteogenic differentiation factors into the culture medium, thereby promoting in vitro pre-osteogenic stimulation of stem cells. Furthermore, the membrane enables the release of cytokines by the co-cultured encapsulated cells (Correia et al., Sci Rep., 2016, 6 (1), 21883; Nadine et al., Biofabrication, 2019, 12 (1), 15005). In this current study, we evaluated indicative factors that confirm the membrane's capability to facilitate nutrient diffusion and waste product exchange. This assessment was carried out by quantifying the metabolic activity of the encapsulated cells using the MTS assay and by examining the presence and distribution of osteogenic markers by immunostainings. Finally, since the capsules are not obtained in minutes, it is not possible to obtain an accurate result by encapsulating fluorescent probes, since they would start diffusion before the final assembly of the multilayered membrane.

**Comment 5. **Size Distribution of Spheres: Additional images showing the size distribution of the spheres are necessary to evaluate the reproducibility of the fabrication process.

**Response 5.** We acknowledge the reviewer's concern regarding the reproducibility of the fabrication process of the spherical microparticles.  To evaluate the reproducibility of the fabrication process, we measured the diameter of 50 spherical microparticles within the range of 80 to 100 µm and added a graph to **Figure 2C. **The size distribution of spheres 25-40 µm is already described in a previous article from the group (Bjørge et al., Nanoscale, 2019,11, 16214-16221).

**Comment 6.** DNA Quantification Standards: The use of Lambda DNA as a standard for mammalian DNA quantification is suboptimal. The authors are advised to consider calf thymus DNA standards (ThermoFisher, Ref. 15633019) for more accurate quantification.

**Response 6.** We thank the reviewer for this comment. We will consider this suggestion for future works.

**Comment 7.** Metabolic Activity Data (Figure 4B): There is an absence of metabolic activity data for day 3 in Figure 4B. The authors should provide an explanation for this omission.

**Response 7. **We thank the reviewer for pointing out this important mistake.  To address the absence of metabolic activity data for day 3 in **Figure 4B**, we have included an additional graph showing metabolic activity normalized by DNA content, which now includes the data for day 3. This provides a more comprehensive view of the data and ensures that the initial time point is adequately represented in our analysis. We also made all the necessary modifications to the manuscript to accommodate these changes.  

**Comment 8.** SEM Image Clarity (Figure 4C):  The arrows indicating cells in the SEM images are difficult to discern. Profiling with a dotted line would enhance visibility. Additionally, have the authors examined the inner content of the capsules via SEM by sectioning them? The claim of higher ECM density is not convincingly supported by the SEM images provided. Further comments or additional images are required.

**Response 8.** We acknowledge the reviewer’s comment. As suggested the arrows of the SEM images were changed to dotted circumferences to enhance the visibility of the image. We agree that the description of the materials and methods can misguide the interpretation of the results, for the SEM images the capsules were disrupted by mechanical force to analyze the capsules’ content (cells and microparticles). This information has already been modified in the methods section. We agree that the claim of higher ECM density is not convincingly supported by the SEM images provided. Therefore, we have removed this statement from the manuscript to maintain consistency and clarity.

**Comment 9.** Improving Immunostaining (Figure 5B): The use of additional blocking biomolecules (e.g., BSA or serum from the secondary antibody host) could potentially enhance the quality of immunostaining.

**Response 9.** We thank the reviewer for her comment. It was used fetal bovine serum (FBS) as blocking agent to block unspecific staining, instead of BSA, as described in the methods section.

**Comment 10.** Controls for Immunostaining (Figure 5B): Immunostaining images of empty capsules, spheres, and microdiscs should be included as controls. Moreover, immunostaining results for day 14 are missing and should be provided.

**Response 10.** We thank the reviewer for her comment. We believe the control of empty capsules would not add relevant information, because their behavior is similar with or without cells, as with microdiscs or spheres. The capsules only possess a slight autofluorescence to the green laser. Moreover, it was not analyzed the immunostainings of day 14 of culture, since these results were used to showcase the increase of the content of osteopontin during the culture period.  

**Comment 11.** Vascular Population Staining: Complementary immunostainings for vascular populations would confirm their presence, which is critical given that the co-culture system is a focal point of the study.

**Response 11.** We acknowledge the reviewer’s comment, and we agree with the suggestion. This will be taken into consideration in future experiments.
